# Association between lesion location and cervical intraepithelial neoplasia

**DOI:** 10.1590/1806-9282.20261109

**Published:** 2026-11-09

**Authors:** Ömer Mert Yavuz, Erkan Şimşek, İbrahim Taşkum, Selcan Sınacı, Ferhat Aslan

**Affiliations:** 1 Gaziantep City Hospital, Department of Obstetrics and Gynecology – Gaziantep, Turkey.; 2 Gaziantep City Hospital, Department of Obstetrics and Gynecology, Division of Gynecologic Oncology – Gaziantep, Turkey.; 3 Gaziantep City Hospital, Department of Obstetrics and Gynecology, Division of Perinatology – Gaziantep, Turkey.

**Keywords:** Uterine cervical dysplasia, Cervical intraepithelial neoplasia, Colposcopy, Human papillomavirus

## Abstract

**OBJECTIVE:**

The aim of this study was to determine the clock-face distribution of cervical lesions in women undergoing colposcopy for high-risk human papillomavirus positivity and to evaluate the association between lesion localization and high-grade cervical lesions (CIN2+; cervical intraepithelial neoplasia grade 2 or higher).

**METHODS:**

This single-center retrospective observational study included 298 women who underwent colposcopy because of high-risk human papillomavirus positivity between January 2024 and March 2026. Demographic characteristics, human papillomavirus genotypes, cervical cytology, colposcopic biopsy localization, and histopathological findings were retrospectively reviewed. Cervical lesions were classified according to the clock-face system and grouped into anterior, right lateral, posterior, and left lateral regions. The primary outcome was the presence of CIN2+.

**RESULTS:**

The mean age was 42.5±9.2 years, and CIN2+ lesions were identified in 54 women (18.1%). Lesions were most frequently located at the 12 and 6 o'clock positions. Compared with the normal/CIN1 group, patients with CIN2+ had a higher frequency of abnormal cytology (48.1 vs. 23.8%; p=0.001) and anterior cervical localization (88.9 vs. 66.8%; p=0.002). In multivariable logistic regression analysis, abnormal cytology (adjusted OR 2.84; 95%CI 1.43–5.63; p=0.003) and anterior cervical localization (adjusted OR 3.67; 95%CI 1.46–9.21; p=0.006) were independently associated with CIN2+.

**CONCLUSION:**

Cervical lesions in high-risk human papillomavirus-positive women showed a nonuniform clock-face distribution. Anterior cervical localization was independently associated with high-grade cervical lesions (CIN2+), suggesting that careful assessment and documentation of lesion location during colposcopy may improve the identification of clinically significant lesions.

## INTRODUCTION

Cervical cancer is the fourth most common cancer among women worldwide and remains a major cause of morbidity and mortality, particularly in low- and middle-income countries^1,2^. Persistent infection with high-risk human papillomavirus (HR-HPV), especially HPV16 and HPV18, is the principal etiological factor in the development of cervical cancer and its precursor lesions^3,4^. In Turkey, the national cervical cancer screening program recommends primary HPV DNA testing every 5 years for women aged 30–65 years, with HPV-positive women referred for cytological triage and colposcopy when indicated^5^.

Colposcopy is the standard diagnostic procedure for evaluating women with abnormal cervical screening results or HR-HPV positivity and enables directed biopsy of suspicious lesions^6,7^. Lesion location is routinely documented using the cervical clock-face system, which standardizes lesion localization and facilitates histopathological correlation and follow-up assessment^8,9^.

Previous studies have shown that cervical precancerous lesions are not uniformly distributed within the transformation zone; however, their topographical distribution remains controversial. While some studies have reported a predominance of lesions in the posterior cervix^10,11^, others have described genotype-specific clustering or no consistent distribution pattern^12–14^. Moreover, studies investigating the relationship between clock-face localization and histopathological severity in HR-HPV-positive women are limited.

Therefore, this study aimed to determine the distribution of cervical lesions according to the cervical clock-face system in HR-HPV-positive women undergoing colposcopy and to investigate its association with high-grade cervical lesions [cervical intraepithelial neoplasia grade 2 or higher (CIN2+)].

## METHODS

### Study design and participants

This single-center retrospective observational study included women who underwent colposcopy because of HR-HPV positivity at the Colposcopy Unit of the Department of Obstetrics and Gynecology, Gaziantep City Hospital, Türkiye, between January 2024 and March 2026. Women aged 30–65 years with available HPV genotyping, cervical cytology, clock-face documentation of biopsy location, and histopathological results were eligible. Patients with incomplete clinical, cytological, virological, or histopathological data, unavailable histopathological results, previous treatment for cervical malignancy, or cervical surgery substantially altering cervical anatomy were excluded. Consecutive eligible patients were included, resulting in a final study population of 298 women. The study was conducted and reported in accordance with the Strengthening the Reporting of Observational Studies in Epidemiology Statement.

### Data collection and definitions

Demographic characteristics, obstetric history, cervical cytology, HPV genotype, biopsy location, and histopathological findings were retrieved from electronic medical records. For descriptive genotype distribution, HPV infections were categorized according to the detected genotype combinations. In comparative analyses, HPV16 positivity, HPV18 positivity, and other HR-HPV positivity were coded as non-mutually exclusive binary variables according to the presence of the respective genotype; therefore, patients with multiple HR-HPV infections could contribute to more than one genotype category. Multiple HR-HPV infection was additionally coded as a separate binary variable indicating the presence of more than one HR-HPV genotype. Cervical cytology was classified according to the Bethesda System. Atypical squamous cells of undetermined significance (ASC-US), low-grade squamous intraepithelial lesion (LSIL), high-grade squamous intraepithelial lesion (HSIL), atypical squamous cells, cannot exclude HSIL (ASC-H), and atypical glandular cells (AGC) were considered abnormal cytology^15^.

### Colposcopy and histopathological evaluation

Colposcopic biopsy locations were documented using the cervical clock-face system with the external cervical os as the center. Colposcopic examinations were performed by multiple colposcopists as part of routine clinical practice. For the purposes of this retrospective analysis, lesion localization was operationally defined according to the documented site(s) of colposcopically directed biopsy. Accordingly, localization represents biopsy-site localization and should not be interpreted as systematic mapping of the entire cervical epithelium. For statistical analysis, lesion locations were grouped into four anatomical regions: anterior (11–1 o'clock), right lateral (2–4 o'clock), posterior (5–7 o'clock), and left lateral (8–10 o'clock). Lesions involving more than one region were classified as multifocal.

Histopathological diagnoses were categorized as normal, CIN1, CIN2, CIN3, adenocarcinoma in situ (AIS), squamous cell carcinoma, or adenocarcinoma^16^. Normal histology and CIN1 were classified as the low-grade group, whereas CIN2 or worse (CIN2+) was considered the primary study outcome.

### Statistical analysis

Statistical analyses were performed using International Business Machines Statistical Package for the Social Sciences (IBM SPSS) Statistics for Windows, Version 29.0 (IBM Corp., Armonk, NY, USA). Data distribution was assessed using the Shapiro-Wilk test. Continuous variables are presented as mean±standard deviation (SD) or median (interquartile range [IQR]), as appropriate, whereas categorical variables are presented as frequencies and percentages. Patients were categorized into normal/CIN1 and CIN2+ groups. Continuous variables were compared using the Student's t-test or Mann-Whitney U test, and categorical variables were compared using the chi-square test or Fisher's exact test, as appropriate. Variables with p<0.10 in the univariable analysis or considered clinically relevant (age, HPV16 positivity, abnormal cytology, anterior localization, and multifocal lesions) were entered into a multivariable logistic regression model. Adjusted odds ratios (ORs) with 95%CIs were calculated. As an exploratory sensitivity analysis, individual clock-face positions were evaluated separately for their association with CIN2+ using Fisher's exact test, and the Holm method was applied to adjust for multiple comparisons across the 12 clock positions. In addition, a sensitivity analysis using CIN3+ (CIN3 or invasive carcinoma) as a more stringent histopathological endpoint was performed to assess the robustness of the primary findings. A two-sided p<0.05 was considered statistically significant.

## RESULTS

A total of 298 women were included in the study. The mean age was 42.5±9.2 years, with a mean gravidity of 3.3±1.7 and parity of 3.2±1.6. Regarding HPV genotype distribution, HPV16 was detected in 104 women (34.9%), HPV18 in 29 (9.7%), and other HR-HPV genotypes in 132 (44.3%). Coinfections included HPV16 and HPV18 in 21 women (7.0%), HPV16 and other HR-HPV genotypes in 11 (3.7%), and HPV18 and other HR-HPV genotypes in one (0.3%). Cervical cytology was negative in 213 women (71.5%); abnormal or other cytological findings included ASC-US in 47 (15.8%), LSIL in 25 (8.4%), HSIL in 7 (2.3%), ASC-H in 4 (1.3%), AGC in one (0.3%), and an unsatisfactory result in one (0.3%). Histopathological examination revealed normal histology in 218 women (73.2%), CIN1 in 26 (8.7%), CIN2 in 15 (5.0%), CIN3 in 38 (12.8%), and squamous cell carcinoma in one (0.3%). No cases of AIS or adenocarcinoma were identified. Overall, CIN2+ was present in 54 women (18.1%).

Cervical lesions were most frequently identified at the 12 o'clock position (n=150), followed by the 6 o'clock (n=119) and 11 o'clock (n=49) positions, whereas the lowest frequency was observed at the 8 o'clock position (n=4). Among the most frequently sampled locations, the proportion of CIN2+ lesions was highest at the 12 o'clock position (28.0%), followed by the 6 o'clock position (23.5%).

In the exploratory analysis of individual clock-face positions, the association observed for the anterior cervical region was predominantly driven by the 12 o'clock position. CIN2+ was identified in 42 of 150 women (28.0%) with a biopsy at the 12 o'clock position, and this association remained statistically significant after adjustment for multiple comparisons using the Holm method (adjusted p<0.001). In contrast, the 11 o'clock (4/49, 8.2%) and 1 o'clock (3/23, 13.0%) positions were not significantly associated with CIN2+ (Figure 1).

**Figure 1:**
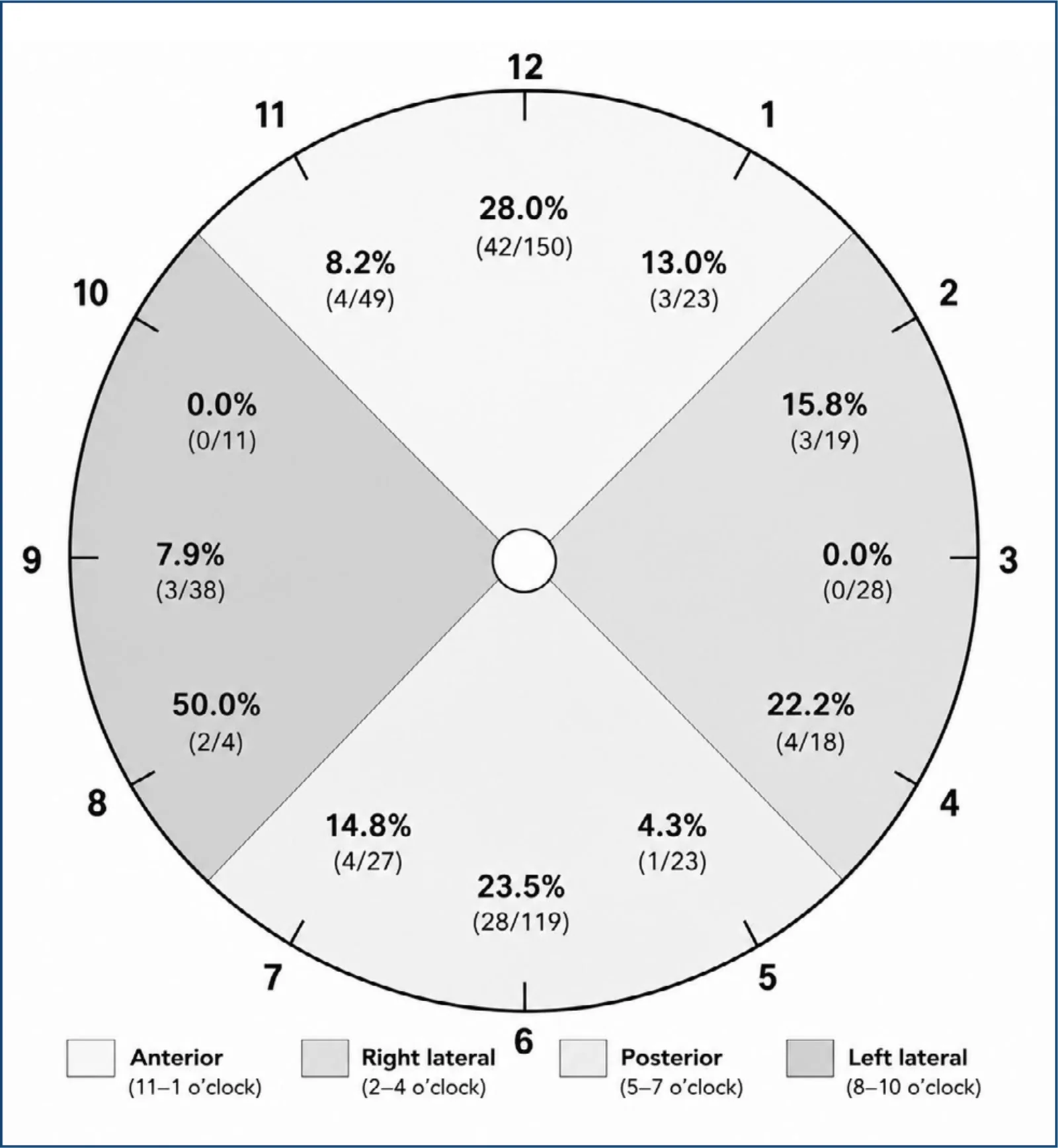
CIN2+ proportions according to cervical clock-face biopsy position. Radial bars represent the proportion of women with CIN2+ at each clock-face biopsy position. Labels indicate the number of CIN2+ cases among women biopsied at the corresponding position, with percentages shown in parentheses. Patients with multifocal lesions may contribute to more than one clock position.

In the sensitivity analysis using CIN3+ as a more stringent histopathological endpoint, anterior cervical localization remained significantly associated with CIN3+ (p=0.014). The association was again predominantly observed at the 12 o'clock position, where CIN3+ was identified in 32 of 150 women (21.3%); this association remained significant after Holm adjustment for multiple comparisons (adjusted p<0.001). The 11 and 1 o'clock positions were not significantly associated with CIN3+.

Patients with CIN2+ had a significantly higher frequency of abnormal cytology than those with normal histology/CIN1 (48.1 vs. 23.8%, p=0.001). Likewise, anterior cervical localization was significantly more common in the CIN2+ group (88.9 vs. 66.8%, p=0.002). No significant differences were observed regarding age, gravidity, parity, HPV18 positivity, multifocal localization, or right, posterior, and left lateral lesion localization. Although HPV16 positivity was more frequent among patients with CIN2+, the difference did not reach statistical significance (57.4 vs. 43.0%, p=0.073) (Table 1).

**Table 1:** Comparison of patients with normal/CIN1 and CIN2+.

Variable	Normal/CIN1 (n=244)	CIN2+ (n=54)	p-value
Age (years)	42.8±9.4	41.3±8.5	0.274
Gravidity	3.2±1.7	3.7±1.8	0.063
Parity	3.1±1.6	3.5±1.7	0.070
HPV16 positivity, n (%)	105 (43.0)	31 (57.4)	0.073
HPV18 positivity, n (%)	42 (17.2)	9 (16.7)	1.000
Other HR-HPV, n (%)	123 (50.4)	21 (38.9)	0.175
Multiple HR-HPV infection, n (%)	26 (10.7)	7 (13.0)	0.796
Abnormal cytology, n (%)	58 (23.8)	26 (48.1)	**0.001**
Multifocal localization, n (%)	143 (58.6)	34 (63.0)	0.662
Anterior localization (11–1 o'clock), n (%)	163 (66.8)	48 (88.9)	**0.002**
Right lateral localization (2–4 o'clock), n (%)	56 (23.0)	6 (11.1)	0.079
Posterior localization (5–7 o'clock), n (%)	131 (53.7)	33 (61.1)	0.400
Left lateral localization (8–10 o'clock), n (%)	49 (20.1)	5 (9.3)	0.094

HPV genotype variables are not mutually exclusive; patients with multiple HR-HPV infections may be represented in more than one genotype category. Therefore, percentages do not sum to 100%. Bold values indicate statistical significance (p<0.05). HR-HPV: high-risk human papillomavirus; CIN: cervical intraepithelial neoplasia.

In the multivariable logistic regression analysis, abnormal cytology (adjusted OR 2.84; 95%CI 1.43–5.63; p=0.003) and anterior cervical localization (adjusted OR 3.67; 95%CI 1.46–9.21; p=0.006) were independently associated with CIN2+. HPV16 positivity showed a trend toward an increased risk but did not reach statistical significance (adjusted OR 1.79; 95%CI 0.95–3.36; p=0.071). Age and multifocal localization were not independently associated with CIN2+ (Table 2).

**Table 2:** Multivariable logistic regression analysis for factors associated with CIN2+.

Variable	OR	95%CI	p-value
Age	0.98	0.94–1.02	0.279
HPV16 positivity	1.79	0.95–3.36	0.071
Abnormal cytology	2.84	1.43–5.63	**0.003**
Anterior localization (11–1 o'clock)	3.67	1.46–9.21	**0.006**
Multifocal localization	1.17	0.58–2.35	0.654

Dependent variable: presence of CIN2+. Odds ratios (ORs) are presented with 95%CIs. Bold values indicate statistical significance (p<0.05). OR: odds ratio; CI: confidence interval; CIN: cervical intraepithelial neoplasia.

## DISCUSSION

In this study, we evaluated the clock-face distribution of cervical lesions and their association with CIN2+ in 298 HR-HPV-positive women undergoing colposcopy. The principal finding was that anterior cervical localization (11–1 o'clock) was independently associated with CIN2+, increasing the odds of high-grade cervical lesions by approximately 3.7-fold. Abnormal cytology was also identified as an independent predictor of CIN2+, whereas HPV16 positivity showed a nonsignificant trend toward an increased risk.

The prevalence of CIN2+ was 18.1%, which is consistent with previous reports among women referred for colposcopy following HR-HPV-positive cervical cancer screening in Turkey^17,18^. This finding reflects a clinically relevant burden of high-grade cervical lesions in this population and further supports the importance of colposcopic evaluation in HR-HPV-positive women.

Previous studies have demonstrated that cervical precancerous lesions are not uniformly distributed across the cervix^10–12^. Zhao et al. reported that CIN2+ lesions exhibit a nonrandom distribution, whereas He et al. observed that high-grade lesions were more frequently located in the posterior cervix despite the 12 o'clock position being the most common biopsy site^10–12^. In our cohort, lesions were most frequently identified at the 12 o'clock position, while anterior localization was independently associated with CIN2+. Notably, the exploratory analysis of individual clock-face positions showed that this anterior association was predominantly driven by the 12 o'clock position, which remained significantly associated with CIN2+ after adjustment for multiple comparisons, whereas the 11 and 1 o'clock positions were not significantly associated with CIN2+. This finding suggests that the observed anterior association may reflect a more localized pattern rather than a uniform increase in CIN2+ across the entire anterior cervical region^10,11^. The robustness of this finding was further supported by the sensitivity analysis using CIN3+ as a more stringent histopathological endpoint. The association with anterior localization remained significant, and the 12 o'clock position continued to show a significant association after correction for multiple comparisons, suggesting that the observed topographical pattern was not solely attributable to the inclusion of CIN2 lesions.

Tamura et al. suggested that the distribution of CIN2/3 lesions may be influenced by HPV status and uterine position, with a wider distribution of CIN2/3 lesions over the anterior cervix in women with an anteverted uterus^13^. However, not all studies have reported a preferential lesion distribution. Lurie et al. found that cervical intraepithelial neoplasia was randomly distributed across the cervix, suggesting that lesion localization may vary according to the study population, biopsy strategy, and colposcopic assessment^14^. These findings indicate that lesion distribution is likely influenced by multiple anatomical and biological factors rather than by clock-face location alone.

Anterior cervical localization remained independently associated with CIN2+ after adjustment for age, HPV16 positivity, abnormal cytology, and multifocal localization. However, this association should be interpreted cautiously. Because lesion localization in the present study was derived from biopsy sites, the observed distribution may have been influenced by differential visualization and biopsy sampling. The anterior cervix may be more readily visualized during colposcopy, potentially increasing the likelihood of targeted biopsy in this region. Alternatively, differences in transformation zone exposure or cervical anatomy may also contribute to the observed distribution. Although Pretorius et al. reported a slightly higher prevalence of CIN2+ in the posterior cervix, they also observed that visible HPV/CIN1 lesions were more commonly detected anteriorly^19^. Therefore, our findings demonstrate an association between anterior biopsy-site localization and CIN2+ but cannot establish a true biological predilection of high-grade lesions for the anterior cervix.

Previous studies have shown that obtaining multiple directed biopsies improves the detection of CIN2+ lesions^19–22^. Therefore, documenting lesion location using the cervical clock-face system may not only facilitate standardized reporting but also contribute to biopsy planning during colposcopic examination. Although our findings highlight the importance of careful assessment of the anterior cervix, they should not be interpreted as supporting preferential biopsy of this region at the expense of a comprehensive colposcopic examination. Rather, systematic documentation of lesion location and thorough evaluation of the entire transformation zone remain essential, while the observed anterior predominance warrants validation in prospective studies using standardized biopsy protocols.

Consistent with previous studies, abnormal cytology was an independent predictor of CIN2+, highlighting the continued value of cytological abnormalities in the clinical triage of HR-HPV-positive women^7,19^. Beyond conventional clinician-based screening pathways, recent studies have explored self-sampling as an alternative approach to HPV testing. Lorenzi et al.^23^ evaluated self-collected vaginal samples in combination with p16/Ki-67 dual staining, while Castanheira et al.^24^ investigated vaginal and urine self-sampling for HPV genotyping in women with high-grade cervical lesions. More recently, Lima et al.^25^ compared self-collected and clinician-collected samples for HPV testing in a meta-analysis. These studies address sampling strategies at an earlier stage of the cervical cancer screening pathway and are therefore methodologically distinct from the present study, which focuses on the anatomical distribution of cervical lesions and their association with CIN2+ among HR-HPV-positive women undergoing colposcopy. Nevertheless, together they reflect the evolving role of HPV-based approaches across different stages of cervical cancer screening and diagnostic evaluation. With regard to the lesion characteristics evaluated in our study, multifocal lesion distribution was not independently associated with CIN2+, suggesting that lesion location may be more informative than lesion multiplicity in predicting histopathological severity.

The strengths of this study include the exclusive enrollment of HR-HPV-positive women, the availability of HPV genotyping and histopathological confirmation, and the use of CIN2+ as a clinically relevant outcome. However, several limitations should be acknowledged. First, this was a retrospective single-center study, which may limit the generalizability of the findings. Importantly, cervical localization was defined according to the sites from which colposcopically directed biopsies were obtained. Consequently, the observed topographical distribution reflects not only the underlying distribution of cervical lesions but also lesion visibility, colposcopic assessment, and biopsy-site selection. This introduces the possibility of detection and sampling bias, particularly at more readily visualized cervical positions. Thus, the observed association should be interpreted as a biopsy-site association rather than definitive evidence of an anatomical predilection for CIN2+. In addition, important colposcopic variables, including transformation zone type, colposcopic impression, lesion size, colposcopist experience, and the number of biopsy samples, were unavailable and therefore could not be included in the analysis. Smoking status, immunosuppression or relevant comorbidities, and previous cervical screening history were also not consistently available in the retrospective records and therefore could not be evaluated as potential confounders. Furthermore, given the limited number of CIN2+ events relative to the number of predictors included in the multivariable model, the possibility of model overfitting cannot be entirely excluded, and the adjusted estimates should therefore be interpreted with caution. Information regarding the use of p16 immunohistochemistry was not consistently available in the retrospective records and therefore could not be incorporated into the analysis. Given the recognized interobserver variability in the histopathological diagnosis of CIN2, this represents an additional limitation; however, the persistence of the main topographical association in the CIN3+ sensitivity analysis provides some reassurance regarding the robustness of the findings. Finally, patients with multifocal lesions contributed to more than one clock-face location; consequently, localization-based analyses were not entirely independent and should be interpreted with caution.

## CONCLUSION

Cervical lesions in HR-HPV-positive women were not uniformly distributed across the cervix, with the highest frequencies observed at the 12 and 6 o'clock positions. Anterior cervical localization and abnormal cytology were identified as independent predictors of CIN2+. These findings suggest that lesion location should be considered alongside colposcopic appearance during the evaluation of HR-HPV-positive women. Larger prospective multicenter studies are warranted to confirm the association between anterior cervical localization and high-grade cervical lesions.

## Data Availability

The datasets generated and/or analyzed during the current study are available from the corresponding author upon reasonable request.
